# Incidence rates and treatment of the transcervical fracture of the neck of femur in Italy: is total hip arthroplasty an increasingly preferred approach? A population study on trends between 2001 and 2023 based on 1,120,770 hospital discharge records

**DOI:** 10.1186/s10195-026-00912-y

**Published:** 2026-03-14

**Authors:** Enrico Ciminello, Emilio Romanini, Michele Venosa, Gianpiero Cazzato, Gabriele Tucci, Filippo Boniforti, Luca Carpanese, Adriano Cuccu, Tiziana Falcone, Paola Ciccarelli, Stefania Ceccarelli, Marina Torre

**Affiliations:** 1 Italian Implantable Prostheses Registry (RIPI), Italian National Institute of Health, Viale Regina Elena 299, 00161 Rome, Italy; 2 RomaPro, Polo Sanitario San Feliciano, Via Mattia Battistini 44, 00167 Rome, Italy; 3 GLOBE, Italian Working Group on Evidence-Based Orthopaedics, Via Nicola Martelli 3, 00197 Rome, Italy; 4 Ospedale dei Castelli, 00040 Ariccia, Italy; 5 Fondazione Istituto G. Giglio, 90015 Cefalù, Italy; 6 Department of Statistical Sciences, Sapienza University of Rome, Piazzale Aldo Moro 5, 00185 Rome, Italy

**Keywords:** Femoral fractures, Femoral head, Fragility fractures, Epidemiology, Trends, Hip arthroplasty

## Abstract

**Introduction:**

Transcervical femoral neck fractures (TFNFs) are among the most devastating fragility fractures in the elderly. TFNF are associated with excess 1-year mortality rates ranging from 15% to 30%. Treatments include conservative methods, internal fixation, and arthroplasty (partial or total hip arthroplasty). This study aims to analyze the changes in incidences of TFNF in the Italian population between 2001 and 2023 and the evolution of the choices of treatment.

**Materials and methods:**

Using hospital discharge record (HDR) data from 2001 to 2023, records with ICD9-CM codes for femoral neck fractures (820.0 and 820.1) among diagnoses were selected and categorized into four treatment groups: total arthroplasty, partial arthroplasty, fixation, and conservative. Time series were analyzed with stratification by sex and age.

**Results:**

The extracted data included 1,120,724 records of TFNFs, with 871,161 cases treated surgically (total or partial arthroplasty or internal fixation) and 249,563 treated conservatively; the average patient age was 79.1 years, with a higher proportion of women (72.8%). Partial hip arthroplasty was the preferred treatment overall. For younger patients, in the age classes < 45 and 45–54 years, fixation was the most chosen treatment. Over time, the use of the conservative treatment decreased from 27.5% in 2001 to 14.6% of cases in 2023. The use of partial and total hip arthroplasty increased from 40% and 13.3% in 2001 to 44.5% and 24.3% in 2023, respectively.

**Conclusions:**

Over the past two decades, Italy experienced declining age-adjusted incidence rates of TFNF despite persistent crude numbers (approximately 50,000 cases per year) owing to demographic aging. Partial hip arthroplasty (PHA) remained the preferred treatment, while total hip arthroplasty (THA) went from being the least used to the second-most performed treatment through the 23 observed years.

*Level of evidence* level 1, population-based study

**Supplementary Information:**

The online version contains supplementary material available at 10.1186/s10195-026-00912-y.

## Introduction

Transcervical femoral neck fractures (TFNFs) are among the most devastating fragility fractures in elderly individuals, often triggering a cascade of functional decline, loss of independence, and excess mortality. Their impact extends far beyond the acute surgical episode, with major social and economic consequences for patients, caregivers, and healthcare systems [1–4]. Globally, the occurrence of TFNF varies by geography, ethnicity, and socioeconomic conditions. High-income countries have reported stable or declining age-adjusted rates over the last 20 years, likely owing to improvements in osteoporosis management, fall prevention strategies, and healthier aging lifestyles [5–9]. However, the global absolute number of fractures is set to increase significantly and almost double by 2050, driven by rapid population aging, particularly in Asia and South America [1, 10]. This trend emphasizes the need for coordinated global and national efforts to mitigate the clinical and economic burden. Clinically, TFNFs are associated with excess 1-year mortality rates ranging from 15% to 30%, depending on patient age, comorbidities, and quality of care [11]. Functional recovery is often incomplete, with many patients never regaining pre-fracture mobility or independence. Early surgery, within 24–48 h, is recommended to reduce complications and mortality, yet access to timely surgical care remains uneven worldwide [12]. Therapeutic options depend on patient age, functional demands, and fracture patterns. Internal fixation is generally preferred for younger patients to preserve the native joint, while arthroplasty—either partial hip arthroplasty (PHA) or total hip arthroplasty (THA)—is standard for displaced TFNF in older adults [13–16]. THA offers better long-term functional outcomes in selected, active older patients but with a higher risk of dislocation and longer operative times compared with PHA [14]. The choice between cemented and uncemented fixation remains clinically relevant, balancing improved initial stability with potential perioperative risks [13].

This study aims to analyze the changes in incidences of TFNF in the Italian population between 2001 and 2023 and the evolution of the choices of treatment in hospitalized patients at the national level, comparing trends of performed THA, PHA, fixations, and conservative approaches. Incidences and treatment differences are also explored after stratification by sex and by age class.

## Materials and methods

This population-based, observational study was carried out according to the Strengthening the Reporting of Observational Studies in Epidemiology (STROBE) guidelines provided by the Enhancing the Quality and Transparency of Health Research (EQUATOR) Network [17] and passed all the steps of the REporting of studies Conducted using Observational Routinely-collected health Data (RECORD) statement [18].

### Data source

Every year, the Italian Ministry of Health consolidates the Hospital Discharge Record (HDR) Database and provides the Italian National Institute of Health with an anonymized version of it. Such a dataset collects information about 94–99% of the hospitalizations that occurred at the national level, with associated diagnoses, performed procedures, and demographic features of patients [19]. Diagnoses and procedures are reported in terms of the International Classification of Diseases, 9th revision—Clinical Modification (ICD9-CM), with a maximum of 6 (1 main + 5 secondary) and 11 (1 main + 10 secondary) associated codes, respectively.

### Study design

A set of 239,560,403 HDRs was browsed, from which records of interest were extracted according to the following rules:

- At least once, an ICD9-CM code with 820.0 or 820.1 appeared as the first four digits (“Fracture of neck of femur: Transcervical fracture closed” or “Fracture of neck of femur: Transcervical fracture open”) among principal or secondary diagnoses.
- Sex and age variables were correctly reported.

Such records were then labeled as “total hip arthroplasty” (THA), “partial hip arthroplasty” (PHA), “fixation” or “conservative,” depending on the ICD9-CM codes reported in principal or secondary procedures. Record extraction and classification were performed according to the following operative flow:

1. IF (81.51) IN procedures THEN record is classified as “total hip arthroplasty.”
2. IF (81.52) IN procedures AND (81.51) NOT IN procedures THEN record is classified as “partial hip arthroplasty.”
3. IF (79.05 OR 79.15 OR 79.25 OR 79.35) IN procedures AND (81.51 OR 81.52) NOT IN procedures THEN record is classified as “fixation.”
4. IF (79.05 AND 79.15 AND 79.25 AND 79.35 AND 81.51 AND 81.52) NOT IN procedures THEN record is classified as “conservative.”

The described rules imposed a hierarchical structure in which records reporting codes for both THA and PHA were classified as THA, records reporting codes for both THA and fixation were classified as THA, and records reporting codes for both PHA and fixation were classified as PHA. Such a hierarchy is based on common clinical sense, as it reflects the reversibility of the surgery: a femur or a hip replaced by a prosthesis cannot be restored as original, while a preserved anatomical component can be afterwards replaced by a prosthesis. Indeed, an ineffective fixation can lead to a PHA (replacing the femur) or a THA, and a PHA can be revised by a THA (replacing the acetabular component after having replaced the femoral one), while the reverse cannot be done.

### Measures

The time series on counts of the performed procedures and incidence rates (IRs) per 100,000 inhabitants were analyzed overall, as well as after stratification by sex and age class independently and by sex and age class combined. Given the variation of age distribution in Italy along the considered time period, and considering that the TFNF affects mostly elderly people, age-adjusted incidence rates (AAIRs), with 2001 as the base year, were computed to provide a sound comparison among different years. Age classes were defined according to the ones used in the Italian Arthroplasty Registry [20].

### Statistical analysis

In each stratum, age was summarized in terms of mean (standard deviation), while counts were reported in terms of counts (percentages). IRs and AAIRs were estimated via a Poisson model, based on census data on the population provided by the Italian National Statistical Institute [21], and reported with 95% confidence intervals (CI_95%_). Age adjustment in AAIRs was computed taking the 2001 Italian population as reference. Variations in IRs and AAIRs between years were reported in terms of estimated incidence rate ratios (IRRs) with CI_95_. The overall dynamics of trends for counts and IRs and AAIRs were tested as increasing or decreasing by using the Cox–Stuart test to check for possible random fluctuations and provide a more comprehensive understanding of the overall behavior of the time series. Proportion changes in treatments over time were explored via the proportion trend test. Significance threshold was fixed at 0.05 for *p*-values. The statistical analysis was performed by using the software R version 4.4.2 (2024-10-31 ucrt) – “Pile of Leaves.”

## Results

**Fig. 1:**
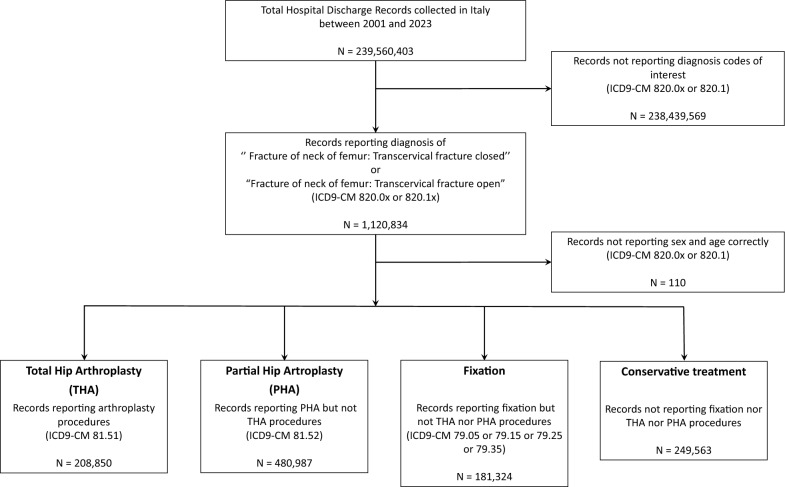
Flowchart for data selection and filtering

After the selection procedure, 1,120,724 records on hospital admissions owing to TFNFs were kept and analyzed (Fig. 1).

TFNF affected patients had a mean age of 79.1 (12.2) years, and were mostly women (815,352 cases; 72.8%). PHA was the preferred treatment on overall, with 480,987 out of 1,120,724 (42.9%) cases being the main way elderly patients—the most affected class (74.7% of patients were over 75 years of age)—were treated. For younger patients, in the age classes < 45 and 45–54 years, fixation was the most chosen treatment, with 12,704 out of 22,128 (57.4%) and 13,085 out of 27,386 (47.8%) cases, respectively. THA was the preferred treatment for patients aged between 55 and 74 years, with 26,313 cases out of 65,524 (40.1%) in the age class 55–65 years and 65,722 cases out of 168,611 (40%) in the age class 65–74 years. Table 1 reports the distributions of sex and age classes within all treatment groups.

**Table 1:** Sex and age distribution within treatment groups

	THA	PHA	Fixation	Conservative	Total
Age, years	74 (10.4)	83.7 (7.3)	73.5 (16.9)	78.8 (13.4)	79.1 (12.2)
Female	155,258 (74.3%)	359,685 (74.8%)	125,050 (69%)	175,359 (70.3%)	815,352 (72.8%)
Male	53,592 (25.7%)	121,302 (25.2%)	56,274 (31%)	74,204 (29.7%)	305,372 (27.2%)
Age < 45 years	1731 (0.8%)	395 (0.1%)	12,704 (7%)	7298 (2.9%)	22,128 (2%)
45 ≤ age < 55 years	7421 (3.6%)	1270 (0.3%)	13,085 (7.2%)	5610 (2.2%)	27,386 (2.4%)
55 ≤ age < 65 years	26,313 (12.6%)	5999 (1.2%)	19,786 (10.9%)	13,426 (5.4%)	65,524 (5.8%)
65 ≤ age < 75 years	65,722 (31.5%)	38,001 (7.9%)	28,306 (15.6%)	36,582 (14.7%)	168,611 (15%)
75 ≤ age < 85 years	77,366 (37%)	201,702 (41.9%)	53,990 (29.8%)	94,974 (38.1%)	428,032 (38.2%)
Age ≥ 85 years	30,297 (14.5%)	233,620 (48.6%)	53,453 (29.5%)	91,673 (36.7%)	409,043 (36.5%)
Total	208,850 (100%)	480,987 (100%)	181,324 (100%)	249,563 (100%)	1,120,724 (100%)

Age: mean (standard deviation). *THA*  total hip arthroplasty, *PH* partial hip arthroplasty

**Fig. 2:**
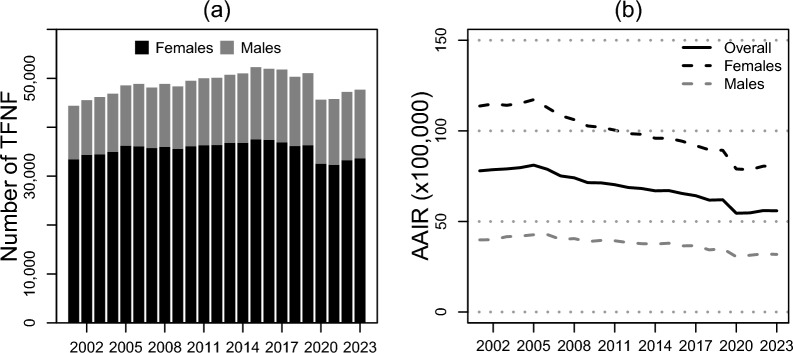
**a** Yearly number of transcervical fracture of the neck of the femur by sex. **b** Age-adjusted incidence rates (× 100,000) of transcervical fracture of the neck of the femur by sex

When looking at the overall trend over the years, the number of hospitalizations involving TFNFs remained relatively stable (*p* = 0.27), shifting from 44,393 cases in 2001 to 47,678 cases in 2023, affecting women in over 70% of the cases every year (Fig. 2a; Supplementary Table A1.1). As reported in Fig. 2b, AAIRs showed a significant decrease (*p* < 0.01) from 77.9 (CI_95%_ 77.2, 78.7) cases per 100,000 inhabitants to 55.9 (CI_95%_ 55.3, 56.5), with an IRR equal to 0.7 (CI_95%_ 0.7, 0.7). The AAIR in women more than doubled the AAIR for men every year, but significantly decreased (*p* < 0.01) over the years, starting from 113.7 (CI_95%_ 112.5, 114.9) cases × 100,000 women in 2001 to 80.7 (CI_95%_ 79.7, 81.7) in 2023, with an IRR equal to 0.7 (CI_95%_ 0.7, 0.7) (Fig. 2).

### Changes in treatment

**Fig. 3:**
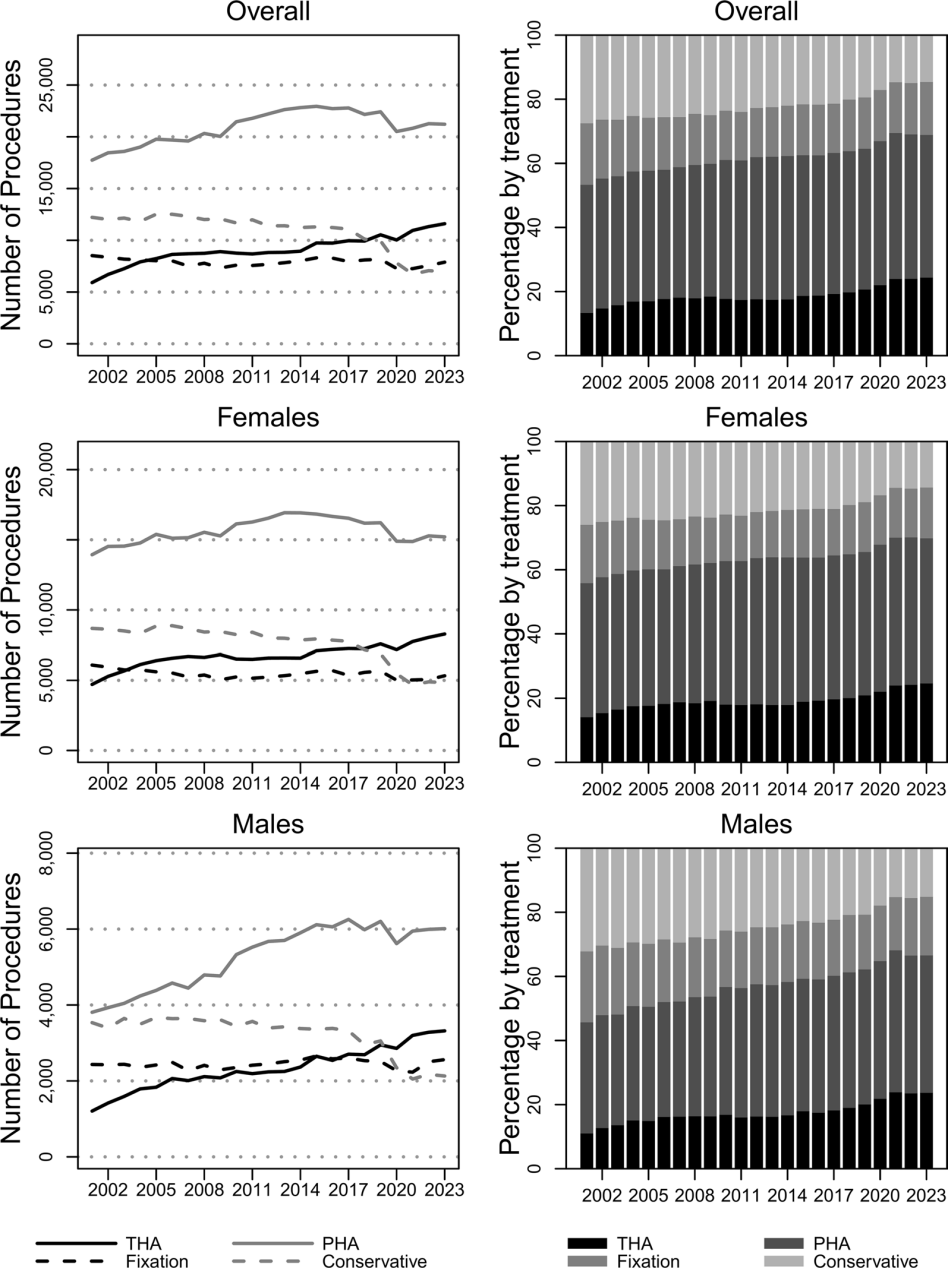
Trends (left column) and proportions (right column) of treatments (by sex) over the years 2001–2023

Figure 3 shows trends of counts and proportions for each treatment (overall and by sex). PHA was always the preferred treatment over the 23 observed years, significantly increasing from 17,743 cases in 2001 to 21,217 cases in 2023 (*p* < 0.05). The use of THA almost doubled over the years (*p* < 0.01), passing from 5913 cases in 2001 to 11,603 cases in 2023. The conservative treatment, used in 12,217 (27.5%) cases in 2001, was decreasingly chosen over the years (*p* < 0.01), reaching 6972 (14.6%) cases in 2023. The overall IR of TFNF increased (*p* = 0.27) from 77.9 (CI_95%_ 77.2, 78.7) cases per 100,000 inhabitants in 2001 to 80.8 (CI_95%_ 80.1, 81.5) in 2023 (IRR 1, CI_95%_ 1, 1.1). PHA was the treatment with the highest IR every year, significantly increasing (*p* < 0.05) from 31.1 (CI_95%_ 30.7, 31.6) cases per 100,000 inhabitants in 2001 to 36.0 (CI_95%_ 35.5, 36.4) cases per 100,000 inhabitants in 2023 (IRR 1.2, CI_95%_ 1.1, 1.2). The IR of THA almost doubled over the 23 observed years (*p* < 0.01), shifting from 10.4 (CI_95%_ 10.1, 10.6) to 19.7 (CI_95%_ 19.3, 20.0) cases per 100,000 inhabitants, with an IRR equal to 1.9 (CI_95%_ 1.8, 2.0).

**Fig. 4:**
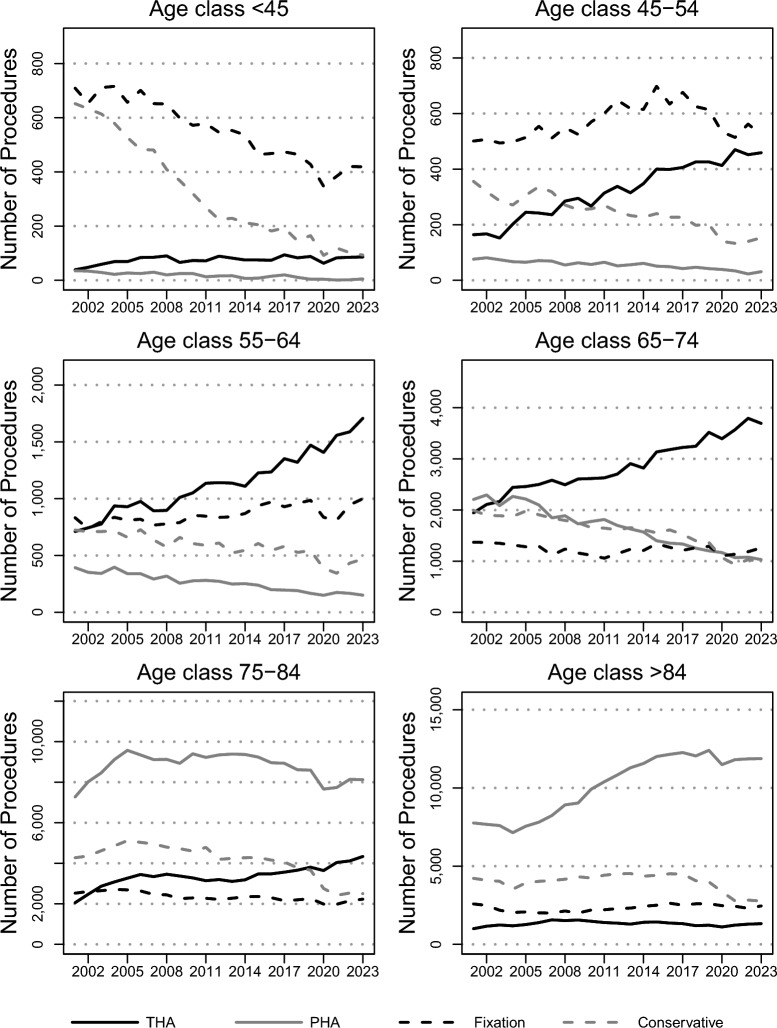
Count trends by treatment and by age class over the years 2001–2023

Figure 4 shows the trends of the treatments over time for each age class. Fixation was the preferred treatment for the two lowest age classes (< 45 years and 45–54 years). THA was the preferred treatment for patients in the 55–64 years and 65–74 years age classes, with an increasing trend (*p* < 0.01) over the 23 observed years in both classes. Lastly, PHA was the preferred choice for patients between 75 and 84 years of age, with THA also increasingly performed (*p* < 0.01) for patients over 85 years. THA significantly increased (*p* < 0.01) until becoming the chosen treatment in more than 50% of cases for patients 55–64 years and for patients 65–74 years, while the conservative treatment was decreasingly used (*p* < 0.01), and in 2023 was chosen in around 15% of cases in all age classes (Supplementary Table A1.2).

The population stratum most affected by TFNF was composed of women over 85 years of age, with 1432.1 (CI_95%_ 1407.0, 1457.5) cases per 100,000 in 2001, decreasing (*p* < 0.01) to 899 (CI_95%_ 883.8, 914.4) cases per 100,000 in 2023, with an IRR equal to 0.6 (CI_95%_ 0.6, 0.6). In general, the TFNF IR for women remained stable in the observed time window, as shown by the IRR being equal to 1.0 (CI_95%_ 1.0, 1.0), with 113.7 (CI_95%_ 112.5, 114.9) cases per 100,000 women in 2001 and 111.5 (CI_95%_ 110.3, 112.7) cases in 2023. Conversely, men were increasingly affected (*p *= 0.01), with IR shifting from 39.8 (CI_95%_ 39.1, 40.6) cases per 100,000 men in 2001 to 48.7 (CI_95%_ 47.9, 49.5) cases per 100,000 in 2023, and an IRR equal to 1.2 (CI_95%_ 1.2, 1.3). When adjusting for population aging dynamics, only the AAIR of THA increased (*p* = 0.03) over the observed period, from 10.4 (CI_95%_ 10.1, 10.6) to 15.0 (CI_95%_ 14.7, 15.3) cases per 100,000, with an IRR equal to 1.4 (CI_95%_ 1.3, 1.4). Supplementary Table A1.4 reports AAIRs by sex and by age class, as well as combined.

Supplementary Tables A1.1, A1.2, and A1.3 report all figures, with IRRs and CI_95%_ values, when computable, of counts, proportions, and IR time series, respectively, as well as by sex and by age class independently and combined. Supplementary Table A1.4 reports AAIRs by sex and for overall data.

## Discussion

This study provides a complete overview of the impact of TFNFs on the Italian population by the exploration of incidences by sex and age, along with the changes in clinical practice to address this issue over 23 years, by presenting figures at the population level. TFNFs affected over 45,000 patients per year, mainly women (74.3% of cases) and people over 75 years of age (74.7%). The overall incidence of TFNF remained stable over the observed period, but when adjusting for age, a significant decrease in the estimated incidence from 77.9 (CI_95%_ 77.2, 78.7) cases per 100,000 inhabitants to 55.9 (CI_95%_ 55.3, 56.5) was observed. The combined observation of crude IRs and age-adjusted incidence rates (AAIRs) highlighted that this might be because of a combined effect of population aging (with the consequence of having more people at high risk of TFNF) and a reduced individual risk of TFNF events, even in the elderly. The reduction of the risk of TFNF might be the result of effective prevention practices, such as changes in lifestyle, eating habits, and pharmacological treatment of osteoporosis, promoted in the last decades [22].

The preferred treatment was PHA along the entire explored time window, with an increasing use of THA, in particular among patients between 55 and 74 years of age. This pattern recalls what was found in the Italian population for the treatment of proximal humeral fractures, where the use of total arthroplasty has more than quadrupled in patients between 55 and 84 years of age in the last two decades [23]. This might confirm an increasing reliability in terms of efficacy and safety of devices and surgical techniques in total arthroplasty for the treatment of major joint fractures. Fixation was mainly used to treat patients under the age of 55 years. The data show that the burden of TFNF on the healthcare system remained unchanged over the years in terms of people affected by this issue.

While our findings of level crude incidence rates, alongside a notable decline in AAIRs, are typical in trends for most high-income nations, the Italian context—situated in the low-incidence Mediterranean cluster—provides valuable insights on how healthcare organization, clinical practice, and demographic pressures assert themselves in TFNF burden. By systematically comparing these results with US data, the rest of Mediterranean European systems, the UK, Scandinavian countries, and Australia, we may be able to interpret the underlying mechanisms of observed trends, better understand the structural determinants of outcomes, and estimate the generalizability of our results [24–26].

In the USA, long-term cohort data of the Framingham Heart Study [27] showed a 4.4% decrease per annum in age-adjusted incidence of hip fractures from 1970 to 2010, which was related to healthier lifestyles, more physical activity, and better osteoporosis treatment. Technically, US management of displaced TFNF in the elderly is predominantly arthroplasty with a relatively higher percentage of cementless fixation compared with Europe. Implant cost matrices, training tradition, and perioperative anesthesia practice variation are some of the factors that influence this [28]. Australia’s rate of hip fractures has experienced a moderate reduction in age-standardized analysis, but demographic aging is driving a rise in the absolute number [29]. While the Australian Orthopaedic Association National Joint Replacement Registry (AOANJRR) conducts comprehensive arthroplasty surveillance, specific data on hip fractures are not collected by the AOANJRR [30]. Technically, the use of implants is more diverse in Australia: cemented stems remain common among the elderly, but uncemented constructs are used in selected patients according to surgeon preference and payment arrangements. The National Hip Fracture Database (NHFD) of the UK is a model of how a national, compulsory clinical audit can induce care improvement. Since its creation in 2007, the NHFD has collected detailed data for all admissions for hip fracture, such as time-to-surgery, orthogeriatric assessment, bone health assessment, and early mobilization. The link between audit participation and financial reward through the Best Practice Tariff has engendered measurable gains: increasing rates of 36-h surgery, improved rates of orthogeriatric input, and reductions in 30-day mortality [31]. Technically, UK practice is characterized by the prevalent use of cemented hemiarthroplasty in elderly patients with displaced TFNF, supported by randomized controlled trials and evidence from the National Joint-Registry showing lower revision rates and better functional scores compared with uncemented styles [32]. Furthermore, the UK has shown one of the highest rates of initiation of secondary prevention of fractures following hip fracture (> 50%) through predominantly integrated Fracture liaison service (FLS) in both acute and community environments [33]. Denmark, Norway, Sweden, and Finland have the world’s highest rates of hip fracture occurrence, with the best registry system and quality improvement processes in the world. The Danish Multidisciplinary Hip Fracture Registry (DMHFR) has 100% national coverage with reporting process data (time-to-surgery, type of anesthesia, mobilization timing) as well as outcomes (30-day survival, readmission, reoperation). Analysis within DMHFR found that mobilization within 24-h post-surgery was associated with significantly reduced rates of mortality and readmission [34].

Scandinavian countries also have a higher propensity to perform THA on active older patients, often to the age of 80 years, in line with functioning gains and acceptable rates of complication as reported in registry data. Cemented fixation is most prevalent, underpinned by several decades of survivorship experience in the Nordic Arthroplasty Register Association [35]. They demonstrate that high incidence does not preclude high-quality outcomes if supported by robust data systems and consistent clinical pathways. Also in the USA, a decrease in the incidence of the TFNF was observed, mainly treated by PHA, in particular among the elderly, with an increased use of THA [36–38].

Those patterns adhere to the recommended procedures found in literature. In general, fixation, PHA, and THA are the recommended practices to treat TFNF, with PHA indicated mainly in the elderly and for patients with limited self-sufficiency and physical activity. THA is considered the treatment ensuring the best outcomes in restoring mobility and early functionality, even though it may lead to a slightly higher risk of dislocation and instability. Defining the best treatment option is impossible, as it depends on several peri-operative, clinical, personal, and demographic factors related to the patient. Position and type of fracture, clinical information, health status, as well as age, sex, daily activities, and habits of the patient contribute to determine the most suitable approach depending on the right trade-off between health-related risks (infection, excessive blood loss, bone fragility, repeated fracture, and many others) and restored health and functionality [39–41].

The Italian pattern—characterized by a uniform reduction of age-adjusted rates and steady crude numbers—is emblematic of the interplay between epidemiologic trend and demographic forces. Our data support that in settings where preventive interventions, osteoporosis treatment, and prevention of falls appear to reduce risk at the individual level, the overall number of hip fractures may fail to decline or actually rise as population aging becomes more intense. This “demographic offset” phenomenon, observed in many high-income nations, underpins the notion that clinic-level progress must be complemented with capacity planning at a systems level. Relative to other Mediterranean countries, our findings are consistent with experience elsewhere that has more limited baseline incidence but equivalent problems with completeness and consistency of care pathways. Conversely, high-incidence systems such as Scandinavia and the UK demonstrate that sound registry infrastructure, perioperative workflow standardization, and population adoption of orthogeriatric co-management can yield outstanding short-term and functional outcomes even in populations with greater inherent fracture risk. The transferability of these models to the Italian context is heartening but will require careful reorganization of administration systems, fund allocation, and cultural norms for rehabilitation and long-term care. More broadly, these trends are set to be applied to other aging nations with intermediate fracture rates but few centralized data collection systems, such as some Southern European and parts of the Asia–Pacific health contexts. However, caution should be exercised with extrapolation to populations with very dissimilar epidemiological profiles, healthcare financing models, or social determinants of risk of falling. Ultimately, our results support an integrated approach that weighs prevention, emergency surgical intervention, organized rehabilitation, and robust systems of surveillance in order to preserve gains achieved in reducing the age-adjusted risk of fractures while mitigating the intractable impact of demographic change.

The main limitation of this study is the administrative nature of the data source. Nonetheless, HDRs are a useful source of information with coverage up to 99% [19] and have been proven to correctly report femoral fractures and associated arthroplasties in over 95% of cases [42]. Second, reported diagnoses only highlight the existence of a TFNF, but do not provide measures of the extent of the trauma, making the evaluation of a correct indication to treatment impossible. Last, HDRs do not provide important peri-operative information of clinical relevance about patient, surgical techniques, and medical devices used intra-operatively. Indeed, even if HDRs provide full coverage at the national level, this tool was not specifically built for surveillance in the investigated field, and the collected information might miss or hide crucial features that are specifically highlighted by other registries, even with lower coverage. For this reason, while reliable in terms of magnitude, some details on the type of fracture and the treatment (used device, peri-operative features, surgical technique) cannot be investigated by HDRs alone, without a proper registry such as the ones existing in other countries. Those limitations highlight the importance of mandatory participation for regions and hospitals in the Italian Arthroplasty Registry (RIAP). Indeed, for the time being, participation in RIAP is still voluntary and completeness and coverage only reach 40% and are not close to the most important registries at the international level [43]. The proper feeding of RIAP with high levels of coverage, completeness, and quality of data would allow for collecting crucial information to enhance clinical practice, as RIAP was specifically designed to collect relevant information on arthroplasties and monitor safety and efficacy of implanted prosthetic devices. A possible future extension of the data collection to other implantable devices in orthopedics, such as the tools for internal fixation, may allow the exploration of the topic of treatments for TFNF in further detail, including outcomes and risk factors.

## Conclusions

Over more than two decades, Italy experienced declining age-adjusted incidence rates of TFNF despite persistent crude numbers owing to demographic aging. The trend is consistent with broader global trends in most high-income nations and shows that better osteoporosis care, fall prevention, and acute fracture treatment have started to reduce individual risk levels [44, 45]. Alignment of national practice with the best evidence-based standards could not only enhance clinical outcomes but also ensure sustainable provision of healthcare with the projected global rise in hip fractures.

## Supplementary Information

Supplementary material 1.

## Data Availability

The data that support the findings of this study are available from the Italian Ministry of Health but restrictions apply to the availability of these data, which were used under license for the current study, and so are not publicly available. Aggregated data that support the findings of this study are however available in the Supplementary Materials. Further aggregated data may be available from the authors upon reasonable request.

## Abbreviations

TFNF: Transcervical femoral neck fracture
PHA: Partial hip arthroplasty
THA: Total hip arthroplasty
HDR: Hospital discharge record
ICD9-CM: International Classification of Diseases, 9th revision Clinical Modification
IR: Incidence rates
AAIR: Age-adjusted incidence rates
CI_95%_: 95% confidence intervals
IRR: Incidence rate ratio
RIAP: Italian arthroplasty registry
